# A novel *FLNA* variant in a fetus with skeletal dysplasia

**DOI:** 10.1038/s41439-022-00224-7

**Published:** 2022-12-13

**Authors:** Kyoko Oshina, Yoshimasa Kamei, Asuka Hori, Fuyuki Hasegawa, Kosuke Taniguchi, Ohsuke Migita, Atsuo Itakura, Kenichiro Hata

**Affiliations:** 1 Department of Maternal-Fetal Biology, National Research Institute for Child Health and Development, Tokyo, Japan; 2 Department of Obstetrics and Gynecology, Juntendo University Faculty of Medicine, Tokyo, Japan; 3 Department of Obstetrics and Gynecology, Saitama Medical University Hospital, Saitama, Japan; 4 Center for Maternal-Fetal, Neonatal and Reproductive Medicine, National Center for Child Health and Development, Tokyo, Japan; 5 Department of Laboratory Medicine, St. Marianna University, School of Medicine, Kanagawa, Japan; 6 Department of Human Molecular Genetics, Gunma University Graduate School of Medicine, Gunma, Japan

**Keywords:** Disease genetics, Diseases

## Abstract

Otopalatodigital spectrum disorder (OPDSD) is characterized by variable phenotypes, including skeletal dysplasia, and is caused by pathogenic variants in filamin A-encoding *FLNA*. *FLNA* variants associated with lethal OPDSD primarily alter the CH2 subdomain of the ABD of FLNA. Herein, we report a novel *FLNA* mutation in a fetus with severe skeletal dysplasia in a pregnant multigravida female with a history of repeated miscarriages and terminations.

Otopalatodigital spectrum disorder (OPDSD) is an X-linked inherited condition caused by pathogenic variants in filamin A-encoding *FLNA*. The X-linked OPDSD, characterized by skeletal malformations, includes otopalatodigital syndrome type 1 (OPD1) and type 2 (OPD2), frontometaphyseal dysplasia type 1, Melnick-Needles syndrome and terminal osseous dysplasia with pigmentary skin defects. In OPD2, most affected males die *in utero* or during early life owing to heart failure and coagulopathy^1^. Patients with OPDSD have variable skeletal dysplasia accompanied by brain malformations, cleft palate, cardiac anomalies, omphalocele, and obstructive uropathy^2^. Owing to the variable manifestations of OPDSD, a definitive diagnosis may often be delayed.

FLNA, previously known as the actin-binding 280 kDa protein, contains an N-terminal actin-binding domain (ABD) composed of two calponin homology domains, CH1 and CH2, and two ROD domains followed by 24 Ig repeats^3,4^. FLNA plays a critical role in cellular motility and signaling by cross-linking actin filaments, tethering membrane glycoproteins, and serving as a scaffold for signaling intermediates and has been associated with a wide spectrum of conditions^5^. Considering the overlapping phenotypes, the location of the *FLNA* mutation is helpful to refine the diagnosis. Pathogenic variants leading to OPD2, which is classified as a severe condition among OPDSDs, are primarily localized in CH2 and result in gain-of-function mutations that increase the actin-binding affinity^6,7^.

A 20-week fetus with severe skeletal dysplasia was terminated. The 38-year-old multigravida female who carried the affected fetus had a history of two abortions and two first-trimester miscarriages (Fig. 1a). Her second fetus had shortened limbs and a giant bladder (Fig. 1a, II-2). During this pregnancy, antenatal ultrasound examination revealed ventriculomegaly, shortening of long bones, abnormal digits, cleft palate, low-set ears, omphalocele, imperforate anus, and bilateral hydronephrosis at 16 weeks, leading to a strong suspicion of lethal skeletal dysplasia.

**Fig. 1:**
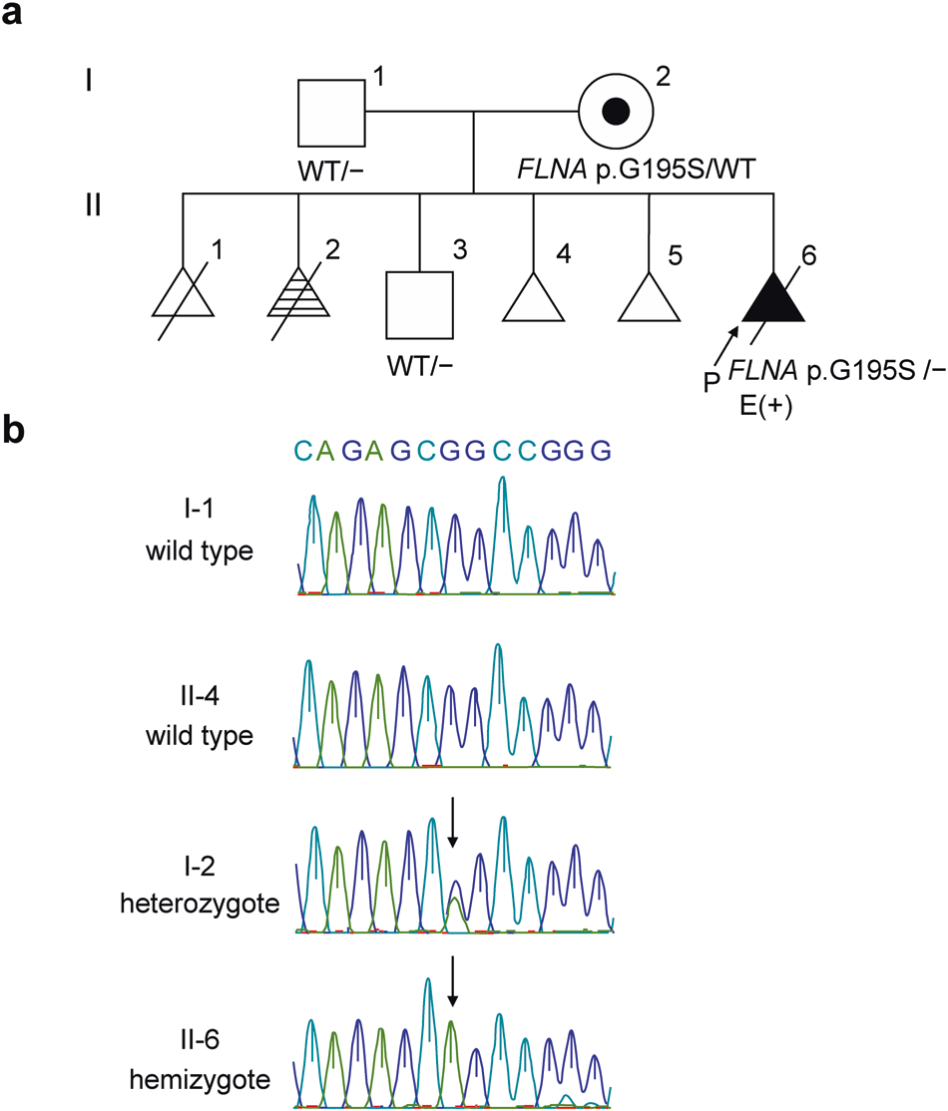
Patient details and the novel missense *FLNA* variant. **a** The patient’s family pedigree. The shaded line represents the case with skeletal dysplasia. Dotted circles represent heterozygous females. **b** Sanger sequencing of wild-type individuals (I-1, II-3), heterozygous females (Patient I-2), and a hemizygous male (Patient II-6).

Under approval from the Ethics Committee of the National Center for Children Health and Development, Tokyo, Japan (IRB number: 926), we sequenced the genomic DNA of I-1, I-2, II-3, and II-6 (Fig. 1a). Whole-exome libraries were prepared using the Agilent SureSelect v6 Capture Kit (Agilent Technologies, Santa Clara, CA, USA) according to the manufacturer’s instructions. The HiSeq 2500 platform (Illumina, Inc., San Diego, CA, USA) was used to perform 150-bp paired-end sequencing. Variant frequencies were obtained from the 1000 Genomes Project database (http://www.internationalgenome.org), Human Genome Variation Database (HGVD; http://www.hgvd.genome.med.kyoto-u.ac.jp), and Tohoku Medical Megabank Organization (ToMMo; https://www.megabank.tohoku.ac.jp). After the selection of variants from the database, the associated phenotypes were obtained from the OMIM database (#305620, #309350, #311300, #304120) for comparison with the actual phenotypes.

Whole-exome sequencing detected a novel hemizygous *FLNA* variant [c.583G > A (p.G195S)] in the region encoding the CH2 subdomain of the ABD of FLNA. This variant has not been registered in variant databases in general populations or reported in individuals with FLNA-related conditions. However, it is predicted to be damaging by the protein function prediction software SIFT (score 0: damaging), PolyPhen-2 (score 1: deleterious), and CADD (score 34). The pathogenic variant-harboring domain is highly conserved, and the substitution of glycine with serine causes a significant physicochemical difference. We assessed the variant according to the ACMG guidelines and classified it as a variant of uncertain significance based on the criteria PM2 (evidence in population database), PP3 (computational evidence), and PP4 (phenotype specific for gene). This potentially deleterious variant was verified by Sanger sequencing, and it was assumed that it had been inherited by the fetus from the mother, who is a heterozygous carrier of this variant (Fig. 1b).

Most *FLNA* variants that lead to OPD2 development have been mapped to the region encoding the CH2 subdomain of the ABD of FLNA (Fig. 2a)^7,8^. Previous reports showed that OPDSD patients with severe phenotypes associated with lethal, prenatal, and neonatal death often have *FLNA* missense mutations in the region encoding this subdomain (Fig. 2b)^1,8–16^. Since only a few functional studies on lethal *FLNA* variants have been conducted, the pathophysiology of OPDSD should be elucidated using a large number of cases. The novel mutation in *FLNA* in the observed patient might be responsible for their severe phenotype. Therefore, it is assumed that the previous miscarriages were caused by the *FLNA* variant, which is often lethal in males, as *FLNA* plays a critical role in human embryonic development^17^. Early diagnosis will provide prospective parents with appropriate genetic counseling services regarding future pregnancies.

**Fig. 2:**
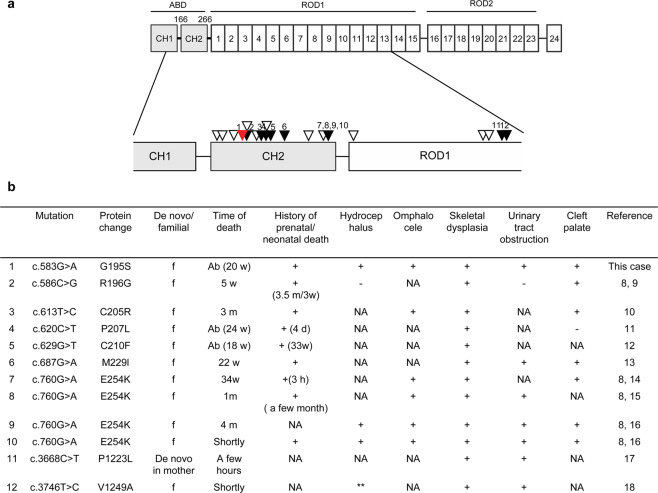
*FLNA* variants with lethal outcomes. **a** Schematic representation of the missense *FLNA* variants in males in the region encoding the concerned protein domains. The red arrowhead indicates the case, and the black arrowhead indicates the lethal outcomes. **b** Clinical characterization and pathogenesis of the *FLNA* variants identified in nonsurviving males.

## Data Availability

The relevant data from this Data Report are hosted at the Human Genome Variation Database at 10.6084/m9.figshare.hgv.3249.
